# Comparative *N*-Glycoproteomics Reveals Subtype-Specific *N*-Glycosylation Signatures and Immune Associations in Cholangiocarcinoma

**DOI:** 10.1016/j.mcpro.2025.101084

**Published:** 2025-10-07

**Authors:** Zhili Xia, Li Gao, Meng Hu, Yingjie Li, Kexin Yu, Ningzu Jiang, Long Gao, Yu Liu, Ying Lu, Yanxian Ren, Chenjun Tian, Yawen Lu, Jindu Zhang, Haiying Yu, Ping Yue, Yanyan Lin, Rou Zhang, Yanqiu Gong, Wenbo Meng

**Affiliations:** 1 The First School of Clinical Medicine, Lanzhou University, Lanzhou, China; 2 National Clinical Research Center for Geriatrics, State Key Laboratory of Biotherapy, West China Hospital, Sichuan University, Chengdu, China; 3 Department of General Surgery, the First Hospital of Lanzhou University, Lanzhou, China; 4 Gansu Province Key Laboratory of Biological Therapy and Regenerative Medicine Transformation, Lanzhou, China

**Keywords:** extrahepatic cholangiocarcinoma, intrahepatic cholangiocarcinoma, *N*-glycoproteomics, *N*-glycosylation, tumor microenvironment

## Abstract

Cholangiocarcinoma (CCA) comprises intrahepatic (iCCA) and extrahepatic (eCCA) subtypes, each exhibiting distinct molecular characteristics. Understanding these differences is critical for identifying subtype-specific therapeutic targets and advancing precision medicine. Protein glycosylation, a key post-translational modification, regulates immune evasion and metastasis, yet the glycoproteomic difference between iCCA and eCCA remains unexplored. Here we presented the first comprehensive *N*-glycoproteomic profile of eCCA and compared it with iCCA using a publicly available dataset. Our *N*-glycoproteomic analysis of paired eCCA tumors and normal adjacent tissues (NATs) identified 8372 *N*-glycopeptides, 3467 *N*-glycosites, and 2627 *N*-glycoproteins. Comparative analysis revealed distinct *N*-glycosylation signature, with eCCA exhibiting higher fucosylated glycans and iCCA showing increased sialylation. Pathway enrichment analysis of *N*-glycoproteins revealed a more prominent lysosome-related enrichment in eCCA, whereas pathways related to immune modulation, cytoskeletal components, and the extracellular matrix were significantly enriched in both subtypes. Immune profiling revealed an immunosuppressive microenvironment in both eCCA and iCCA, characterized by reduced natural killer cell infiltration and subtype-specific fibroblast and endothelial cell remodeling. DPM1, a glycosylation enzyme highly expressed in eCCA, was associated with tumor-specific N-glycopeptides and reduced immune cell infiltration. Its knockdown impaired cell migration, and glycoproteomic analysis implicated DPM1 in regulating adhesion, proteostasis, and immune pathways, highlighting its potential as a therapeutic target in eCCA. Our findings provide insights into *N*-glycosylation alterations in CCA subtypes, underscoring *N*-glycosylation-related mechanisms as potential biomarkers and therapeutic targets, particularly in eCCA.

## Highlights

• Provide a comprehensive *N*-glycoproteomic landscape of eCCA.
• Identify divergent glycosylation signatures between eCCA and iCCA subtypes.
• Disclose an immunosuppressive tumor microenvironment in both eCCA and iCCA.
• DPM1-associated *N*-glycosylation in eCCA is potentially linked to immunosuppression.

## Graphical Abstract

**Figure:**
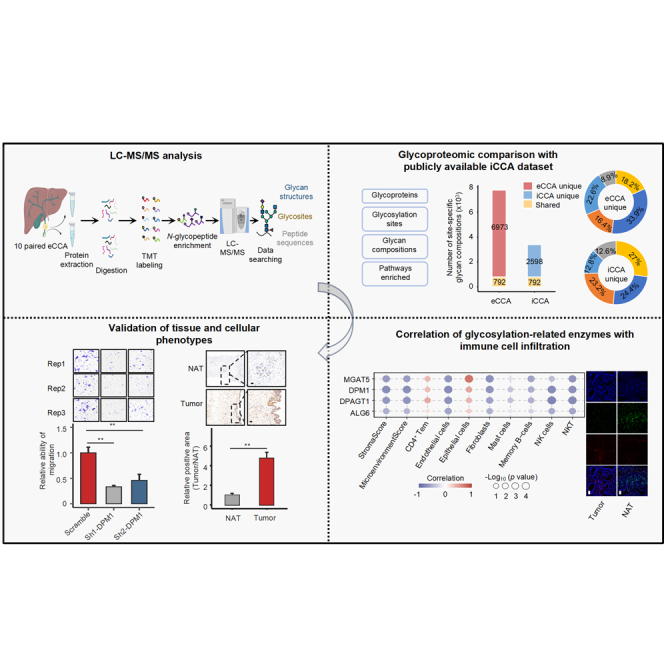

## In Brief

Protein glycosylation plays key roles in the pathogenesis of CCA, yet subtype-specific glycoproteomic features remain largely unexplored. In this study, we established a global *N*-glycoproteomic atlas of eCCA using a TMT-based quantitative strategy and performed a comparative analysis with public iCCA datasets. We identified divergent glycosylation features characteristic of eCCA and iCCA, respectively. In addition, we uncovered several glycosylation-related enzymes and *N*-glycopeptides that were associated with immune cell infiltration in eCCA. These findings highlight immune-associated *N*-glycoproteomic features that may underlie the unique tumor microenvironment of eCCA.

Cholangiocarcinoma (CCA), a highly heterogeneous malignancy originating from the biliary epithelium, is classified into intrahepatic cholangiocarcinoma (iCCA), perihilar cholangiocarcinoma (pCCA), and distal cholangiocarcinoma (dCCA) based on anatomical locations (1, 2, 3). CCA has a low incidence but a high mortality rate, contributing to a growing global burden due to its aggressive clinical course, late-stage diagnosis, and limited treatment options (4, 5, 6). Extrahepatic cholangiocarcinoma (eCCA) is a malignant epithelial tumor arising from the bile duct epithelium outside the liver, distal to the second-order bile duct branches. It includes both pCCA and dCCA subtypes. eCCA is distinct from iCCA in terms of mortality, pathogenesis, molecular characteristics, and therapeutic response (2, 4, 7). For example, iCCA typically manifests with a mass-forming growth pattern, whereas eCCA may present with periductal infiltration or intraductal growth. Additionally, iCCA is often associated with viral hepatitis and liver cirrhosis, while eCCA is more commonly linked to chronic cholangitis and liver fluke infections (8). On a molecular level, iCCA frequently harbors *IDH1* mutations and *FGFR2* fusions, whereas eCCA shows a stronger association with aberrations in the ERBB pathway (9).

Glycosylation is one of the most common post-translational modifications (PTMs), catalyzed by glycosyltransferases that attach either single monosaccharide units or complex polysaccharide chains to specific amino acid residues (10, 11, 12, 13). Emerging evidence indicates that glycosylation plays a crucial role in diverse biological processes, including regulation of protein functions, cell adhesion, and immune evasion (11, 14, 15, 16). In CCA, aberrant glycosylation, including both classical glycan chain modifications and single monosaccharide additions such as O-GlcNAcylation, contributes to multiple oncogenic processes (13, 17, 18). For example, *O*-GlcNAcylation of keratin 18 promotes tumor growth by stabilizing cell cycle regulators and reprogramming cellular metabolism (13), while the glycosyltransferase GALNT5 facilitates CCA progression by enhancing EGFR expression and activation through mucin-type *O*-glycosylation (17). Additionally, terminal fucosylation of exosome-derived β-haptoglobin drives tumor progression and serves as a potential biomarker for diagnosis and prognosis (18). Recent studies have unveiled *N*-glycosylation patterns in iCCA (19, 20), enhancing our understanding of glycosylation in CCA. However, the glycoproteomic features of eCCA, as well as the similarities and differences in the glycosylation landscapes, functions, and clinical relevance between iCCA and eCCA remain unexplored.

The tumor microenvironment (TME) is a dynamic network in which immune cells (e.g. T cells, NK cells, NKT cells, macrophages, etc.) interact with stromal components such as fibroblasts and endothelial cells (21, 22). Aberrant cell-surface glycosylation in cancer can critically modulate these interactions, promoting immune evasion, impairing immune surveillance, and contributing to resistance to immunotherapy (14, 23, 24, 25). These processes collectively drive tumor progression by facilitating angiogenesis, inflammation, and adaptation to hostile conditions. The immune microenvironment can exert either pro- or anti-tumor effects depending on the balance of immune responses (26, 27, 28). Increasing evidence highlights the pivotal role of glycosylation in shaping immune interactions across various cancers. For example, abnormal B7-H3 *N*-glycosylation driven by FUT8 promotes immunosuppression in triple-negative breast cancer (29). In head and neck squamous cell carcinoma, PD-L2 glycosylation enhances immune evasion and mediates resistance to cetuximab treatment, suggesting a potential therapeutic target to improve anti-EGFR efficacy (30). Moreover, in colorectal cancer, *N*-glycosylation of HHLA2 facilitates immune escape by impairing NK cell–mediated responses (31). In CCA, the immune microenvironment plays a crucial role in tumor progression and resistance to therapy (32, 33, 34, 35). However, the interplay between glycosylation and the immune microenvironment in CCA remains understudied.

In this work, we profiled the *N*-glycoproteome of eCCA and further compared the *N*-glycosylomic landscape between eCCA and iCCA, identifying subtype-specific *N*-glycosylation signatures. Besides, we disclosed an immunosuppressive TME in both eCCA and iCCA, and revealed *N*-glycosylation signatures are potentially linked to immune modulation in eCCA, offering new insights into the cancer biology of eCCA.

## Experimental Procedures

### Declaration of Helsinki Principles

The study was approved by the Human Research Ethics Committee of the First Hospital of Lanzhou University (approval number: LDYYLL-2025-752), which granted a waiver of informed consent. Paired tumor and adjacent normal tissue samples were obtained from 10 treatment-naïve patients with eCCA and five treatment-naïve patients with iCCA at the First Hospital of Lanzhou University. Comprehensive clinical data for all cases are provided in Supplemental Table S1. Following surgical resection, the tissues were immediately snap-frozen in liquid nitrogen and stored at −80 °C until analysis. All procedures were conducted in accordance with the ethical standards of the Declaration of Helsinki.

### Experimental Design and Statistical Rationale

This study was designed as a discovery-phase clinical proteomics investigation, aiming to characterize global proteomic and *N*-glycoproteomic alterations in extrahepatic cholangiocarcinoma. The differentially expressed proteins and glycopeptides identified in this analysis are considered exploratory candidates that may guide future validation studies.

For quantitative proteomic and *N*-glycoproteomic profiling of extrahepatic cholangiocarcinoma tissues, 20 paired samples (10 tumors and 10 matched NATs) were analyzed. The NATs were defined as macroscopically normal bile duct segments located as far as possible from the tumor, typically proximal or distal to the lesion, and their nonmalignant nature was confirmed by histopathological examination. Unpaired tumor samples were excluded from the study to ensure consistency in comparative analysis. Samples were divided into three TMT batches, each containing a common pooled internal reference. To ensure data quality, only proteins quantified in ≥50% of samples with at least two unique peptides and glycopeptides quantified in ≥50% of samples were included in downstream analyses. No missing value imputation was performed—differential expression analysis was conducted using the available normalized quantification values.

Differential expression analysis of eCCA used Student’s *t*-tests to compare tumors *versus* NATs. Proteins were deemed significantly altered in eCCA if the tumor/NAT fold change >1.5 or <0.67 with *p* < 0.05. To identify differentially expressed *N*-glycopeptides while minimizing the confounding effects of protein abundance, we applied the following criteria. Glycopeptides were classified as significantly upregulated if they exhibited a fold change >1.5 (*p* < 0.05) and either (i) a greater fold change than the corresponding protein or (ii) no significant change in protein expression. Conversely, glycopeptides with fold change <0.67 (*p* < 0.05) were also considered upregulated if the associated protein showed an even greater decrease, indicating a relative increase in glycosylation occupancy. Similarly, glycopeptides were defined as significantly downregulated if they showed a fold change <0.67 (*p* < 0.05) and either (i) a greater decrease than the corresponding protein or (ii) no significant change in the protein. In contrast, glycopeptides with increased expression (fold change >1.5, *p* < 0.05) were classified as downregulated if the associated protein exhibited a larger increase, reflecting a relative decrease in glycosylation occupancy. These stringent criteria ensured that the detected glycopeptide alterations reflected changes in glycosylation independent of protein-level fluctuations.

All statistical analyses and data visualizations were performed using R software (version 4.3.1). The key R packages utilized in this study include: tidyverse (data manipulation and visualization), ggplot2 (advanced graphics), ComplexHeatmap (heatmap generation), ggpubr (publication-ready statistical plots), corrplot (correlation matrix visualization), and clusterProfiler (functional enrichment analysis). Additionally, reshape2, dplyr, and stringr were used for data reshaping, cleaning, and string manipulation.

### Protein Extraction and Digestion

Tissue samples and cell samples were lysed in RIPA buffer (50 mM Tris-HCl, 150 mM NaCl, 1% NP-40, 0.5% sodium deoxycholate, 0.1% SDS; pH 7.5) supplemented with protease and phosphatase inhibitors. Homogenization was performed using a gentleMACS Dissociator (Miltenyi Biotec) with the “Protein_01_01” program. Lysates were sonicated (10 s on/3 s off cycles for 5 min at 227.5 W) and centrifuged at 20,000*g* for 20 min at 4 °C. The supernatant was collected, and protein concentration was measured by BCA assay. For each sample, 50 μg of protein was reduced with 10 mM TCEP at 56 °C for 1 h, alkylated with 20 mM iodoacetamide at room temperature in the dark for 30 min. Proteins were then precipitated using the methanol–chloroform method with a volume ratio of methanol:water:chloroform:sample = 4:1:1:1 (v/v/v/v). The pellet was resuspended in 50 mM triethylammonium bicarbonate buffer and digested overnight at 37 °C with MS-grade trypsin (Promega) at a 1:50 enzyme-to-protein ratio. The digest was heated to 95 °C for 2 min to inactivate trypsin, then peptides were used for isobaric labeling.

### Isobaric Labeling

A tandem mass tag (TMT)-based approach was employed for quantitative *N*-glycoproteomics and proteomics (36). For eCCA, 10 pairs of tumors and NATs were analyzed using three TMT-labeled batches, each including a common reference sample. This reference was generated by combining equal amounts of peptides from all individual samples to ensure balanced representation of both biological conditions. Peptides were labeled using TMT-10plex reagents (Thermo Fisher Scientific) according to the manufacturer’s protocol. The reference sample was consistently labeled with the same TMT channel (TMT-128C) across all batches. All quantitative data were normalized relative to this internal control channel to minimize batch effects and ensure accurate inter-batch comparisons. After quenching with 5% hydroxylamine, paired tumor and NAT peptides were pooled within each batch. For proteomics analysis, 40 μg of peptides per batch were first desalted using a C18 SPE column (CEREX 10 mg, Tecan). The desalted peptides were fractionated into 120 fractions by high-pH reverse-phase HPLC (Shimadzu LC-2030 Plus) at 1 ml/min. Gradient elution was performed with buffer A (98% H_2_O, 2% acetonitrile, pH 10) and buffer B (90% acetonitrile, 10% H_2_O, pH 10). After fractionation, the 120 fractions were pooled into 20 final fractions using a concatenation strategy, then concentrated using a SpeedVac concentrator. The residual TMT-labeled peptides from each batch were individually retained and used for glycopeptide enrichment. For iCCA, five paired tumor and NAT samples were each labeled with TMT 10-plex reagents using 40 μg of peptide per sample and pooled into one batch for *N*-glycopeptide enrichment. TFK1 cell samples (scramble vs. shDPM1, five biological replicates) underwent the same labeling and pooling procedure for glycoproteomic analysis.

### *N*-glycopeptide Enrichment

*N*-glycopeptides were enriched using custom StageTips packed with ZIC-HILIC resin (Merck Millipore; 5 μm, 200 Å) as described previously (37). Peptides were resuspended in loading buffer (80% acetonitrile, 5% TFA). The StageTip was sequentially activated with H_2_O, 0.5 M NaCl, H_2_O, 80% acetonitrile, and 0.1% TFA, then equilibrated with loading buffer. Peptide mixtures were loaded onto the ZIC-HILIC tip and washed with loading buffer. *N*-glycopeptides were eluted with 300 μl of 0.1% TFA, 100 μl of 50 mM NH_4_HCO_3_, and 100 μl of 50% acetonitrile. Eluates were pooled, concentrated by SpeedVac, and desalted with C18 ZipTips before LC–MS/MS analysis.

### LC-MS/MS Analysis

For proteomic profiling of eCCA tissues, desalted peptides were resuspended in buffer A (2% ACN, 0.1% formic acid) and analyzed using a Nano EASY-nLC 1000 system coupled to a Q Exactive Plus mass spectrometer (Thermo Fisher Scientific). Peptide separation was performed over a 105-min gradient at a flow rate of 330 nl/min, with a stepwise increase in Buffer B (80% ACN, 0.1% FA) as follows: 12% to 32% from 0 to 79 min, 32% to 47% from 79 to 95 min, 47% to 100% from 95 to 99 min, and 100% from 99 to 105 min. Data-dependent acquisition was employed, with MS spectra collected across a mass range of 350 to 1600 m/z at a resolution of 70,000 (m/z = 200). The AGC target was set to 3e6, with a maximum ion injection time of 20 ms. MS2 scans were performed using a 0.6 m/z isolation window and a normalized collision energy (NCE) of 30%, while precursor ions with charge states of z = 1, 5 to 8, >8, or unassigned were excluded.

For *N*-glycoproteomic analysis of eCCA tissue and cell samples, enriched peptides were analyzed using an EASY-nanoLC 1200 system coupled to a Q Exactive HF-X mass spectrometer. Peptide separation was carried out with a 120-min gradient at a flow rate of 330 nl/min, using buffer B (80% ACN, 0.1% formic acid) in Buffer A (98% water, 0.1% formic acid). The gradient profile was as follows: 6%–12% buffer B from 0 to 5 min, 12%–30% from 5 to 92 min, 30%–50% from 92 to 108 min, 50%–100% from 108 to 110 min, and maintained at 100% from 110 to 120 min. Data acquisition was performed in positive ion mode under a data-dependent acquisition strategy. Full MS scans were acquired in the Orbitrap mass analyzer over an m/z range of 700 to 2000, with a resolution of 60,000 at m/z 200. The AGC target was set at 3e6, with a maximum ion injection time of 20 ms. The 20 most intense precursor ions were selected for MS/MS analysis, using a 0.6 m/z isolation window and fragmented with stepped normalized collision energies of 20%, 27%, and 31%. For MS/MS scans, the AGC target was set to 1e5, with a resolution of 30,000 and a maximum injection time of 100 ms. Precursors with charges of z = 1, ≥6, or unassigned were excluded from fragmentation, and an intensity threshold of 2e5 was applied for selection. For the *N*-glycoproteomic analysis of iCCA tissue samples, enriched peptides were analyzed on an EASY-nanoLC 1200 system coupled to an Orbitrap Exploris 480 mass spectrometer. Peptide separation was performed using a 90-min gradient at a flow rate of 330 nl/min, employing Buffer B (80% acetonitrile, 0.1% formic acid) in Buffer A (98% water, 0.1% formic acid). Full MS scans were acquired in the Orbitrap over an m/z range of 350 to 2000 at a resolution of 60,000 (at m/z 200). The top 20 most intense precursor ions were selected for MS/MS analysis using a 0.7 m/z isolation window and fragmented by higher-energy collisional dissociation (HCD) with stepped normalized collision energies of 20%, 27%, and 31%. Precursors with charge states of z = 1, z ≥ 8, or unassigned were excluded from fragmentation.

### MS Data Searching

In this proteomics study, experimental data were processed using MaxQuant software (version 1.6). Protein identification was performed against the Swiss-Prot human protein database (July 2019 release, containing 20,413 sequences), following previously established protocols (38). The mass tolerance for precursor ions was set to 10 ppm, while the fragment ion mass tolerance was restricted to 0.02 Da. For modification settings, carbamidomethylation of cysteine was designated as a fixed modification, whereas methionine oxidation and protein *N*-terminal acetylation were set as variable modifications. To ensure data reliability, peptides shorter than six amino acids were excluded, and a maximum of two missed cleavage sites by trypsin was permitted. Additionally, nested peptides were removed from the analysis. Quality control measures included applying a 1% false discovery rate (FDR) threshold at both the protein and peptide levels, retaining only high-confidence identifications. contaminant and reverse hits were removed, and only proteins identified with ≥2 unique peptides were retained. Total protein intensities were batch-normalized (for individual samples and the reference), then protein intensities were calculated as sample-to-reference ratios per batch, log_2_-transformed, and integrated across the three batches. Proteins with missing values in less than 50% of the samples were included in subsequent analyses.

For *N*-glycoproteomics, intact *N*-glycopeptides were identified using GPSeekerPro (39) by searching against a theoretical *N*-glycan database. The intact human *N*-glycopeptide database was constructed by combining the human proteome database with a curated human *N*-glycome database comprising 6709 entries generated via a retrosynthetic strategy (Supplemental Table S2). The search parameters were consistent with those used in our previously published studies (40). To enable accurate mass matching, TMT labeling at the peptide *N*-terminus and lysine residues was set as a fixed modification. Both precursor and fragment ion searches were performed under stringent criteria, including a minimum peptide length of six amino acids, an isotope peak abundance threshold of 20%, a mass tolerance of 20 ppm, and an isotope abundance deviation limit of 50%. Spectral matching required at least 10% of peptide backbone fragment ions and one glycan-specific fragment ion. The false discovery rate (FDR) was controlled at ≤ 1% using a target-decoy strategy with a P score-based threshold. GPSeekerPro annotated *N*-glycan linkages based on established biosynthetic and retrosynthetic principles. Given the potential co-elution of glycopeptides with identical sequences and compositions, glycan annotations were suggested rather than definitively assigned to avoid misinterpretation. The final dataset included intact *N*-glycopeptides identified by their peptide sequences, glycan compositions, and glycosylation sites. For eCCA, glycopeptide intensities were batch-normalized (including both individual samples and the common reference), followed by calculation of sample-to-reference ratios within each batch. These ratios were log_2_-transformed and then integrated across the three batches. For iCCA and TFK1 cell samples, which were analyzed in a single batch respectively, total glycopeptide intensities were normalized across samples, followed by log_2_ transformation for downstream analysis.

### Evaluation of the Immune Microenvironment

Based on previous studies (41), we applied single-sample GSEA (ssGSEA) and xCell to evaluate the infiltration of 23 immune and stromal cell types, the overall StromaScore and MicroenvironmentScore. Gene sets for these analyses were derived from the literature and the xCell database (41, 42). ssGSEA was performed using the GSVA R package to quantify immune and stromal cell abundance in each sample. The xCell algorithm was used to refine cell-type enrichment scores, providing a detailed characterization of the immune microenvironment. For differential analysis, Student’s *t*-tests compared immune scores between groups. A threshold of *p* < 0.05 was considered significant, and immune or stromal cell types with ≥1.25-fold differences were deemed differentially enriched.

### Western Blotting Analysis

Proteins were resolved by 12.5% SDS-PAGE and transferred to PVDF membranes (ISEQ00010, Millipore). Membranes were blocked with 4% nonfat milk in PBST, incubated with primary antibodies (1:2000) at 4 °C overnight, and then with HRP-conjugated secondary antibodies (1:10,000) at room temperature for 1 h. The following antibodies were used: anti-DPM1 (12403-2-AP, Proteintech).

### Cell Culture and Generation of Stable DPM1-Knockdown Cell Lines

The human extrahepatic cholangiocarcinoma cell line TFK1 was maintained in RPMI-1640 medium (Gibco) supplemented with 10% fetal bovine serum (FBS; FSP500, ExCell Bio), 100 U/ml penicillin, and 100 μg/ml streptomycin (15140-122, Gibco) at 37 °C in a humidified atmosphere with 5% CO_2_. To generate stable DPM1-knockdown TFK1 cells, a short hairpin RNA (shRNA) lentiviral system was employed. Two independent shRNA constructs targeting human DPM1 were designed and packaged into lentiviral particles. Following viral transduction, cells were selected using puromycin (ST551, Beyotime Institute of Biotechnology) to establish stable knockdown lines. The sequences of the shRNAs were as follows:

*DPM1*-shRNA1-sense: 5′- CCACAGGAAACTACATCATTA-3′

*DPM1*-shRNA1-antisense: 5′- TAATGATGTAGTTTCCTGTGG-3′

*DPM1*-shRNA2-sense: 5′- TGAATCCAAGTTGGGAGGAAA-3′

*DPM1*-shRNA2-antisense: 5′- TTTCCTCCCAACTTGGATTCA-3′

### Cell Migration Assay

To evaluate the migratory ability of TFK1 cells, a transwell migration assay was performed using 24-well inserts with 8.0 μm pore membranes (353097, Corning Life Sciences). TFK1 cells (2 × 10^5^) suspended in 200 μl of serum-reduced medium (1% FBS) were seeded into the upper chambers, while 600 μl of complete medium containing 20% FBS was added to the lower chambers as a chemoattractant. After 48 h of incubation at 37 °C, cells on the upper surface of the membrane were removed, and the migrated cells on the lower surface were fixed with 4% paraformaldehyde for 30 min at room temperature and stained with 1% (w/v) crystal violet. Images were acquired under a light microscope, and migrated cells were quantified using ImageJ software.

### Immunofluorescence Staining

Formalin-fixed paraffin-embedded (FFPE) tissue blocks, including histologically confirmed eCCA and NATs, were used for immunofluorescence staining, as previously described (43, 44). CD3 and CD4 antibodies were employed to identify total T cells and CD4^+^ T cell subsets, respectively. NK cells were detected using CD56 antibodies. Nuclei were counterstained with 4′,6-diamidino-2-phenylindole (DAPI). Quantification of immunofluorescent signals was performed using QuPath (45), with positive cells identified based on red and green fluorescence signals. The proportion and spatial distribution of infiltrating immune cells were assessed across representative tumor and NAT regions.

## Results

### *N*-Glycoproteomic Landscape of eCCA

**Fig. 1:**
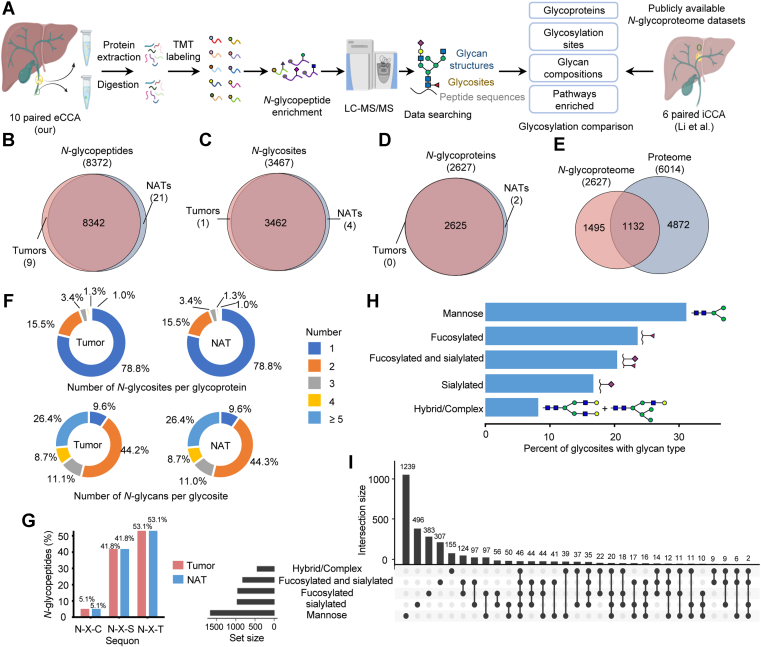
**Characterization of *N*-glycosylation in eCCA.***A*, schematic of t*h*e TMT-based glycoproteomics workflow. Figure created with BioRender.com. *B*-*D*, Venn diagrams of *N*-glycopeptides (*B*), *N*-glycosites (*C*), and *N*-glycoproteins (*D*), showing extensive overlap between tumors and NATs. *E*, Venn diagram showing the overlap between the *N*-glycoproteome and the proteome. *F*. Pie charts illustrating the proportion of *N*-glycoproteins with different numbers of *N*-glycosites per protein (*upper*) and the proportion of *N*-glycosites with varying numbers of *N*-glycans per glycosite in tumors and NATs (*lower*). *G*, distribution of all identified *N*-glycopeptides across N-X-T, N-X-S, and N-X-C tripeptide sequons. *H*, percentage of *N*-glycosites for each glycan types. *I*, UpSet plot showing the frequency of glycan pairs co-occurring at the same *N*-glycosylation site. NAT, normal adjacent tissue; eCCA, extrahepatic cholangiocarcinoma. iCCA, intrahepatic cholangiocarcinoma.

To profile the *N*-glycoproteome of eCCA, we performed TMT-based glycoproteomic analysis of 10 paired tumors and NATs. *N*-glycoproteomic data from six paired iCCA samples were employed for comparative analysis (19) (Fig. 1*A*). Using GPSeekerPro, we identified 8372 *N*-glycopeptides, 3467 *N*-glycosites, and 2627 *N*-glycoproteins in eCCA (Fig. 1, *B–D* and supplemental Table S2). Among the 2627 glycoproteins, 1132 overlapped with the total proteome (Fig. 1*E* and supplemental Table S3). The distribution of glycosites per glycoprotein revealed that the majority (78.8%) of glycoproteins contained a single *N*-glycosylation site, whereas 21.2% had multiple sites (≥2) (Fig. 1*F*, upper panel). Similarly, 9.6% of glycosites were modified by a single glycan composition, whereas 26.4% were modified by more than five distinct glycan compositions, highlighting the site-specific glycosylation heterogeneity in eCCA. (Fig. 1*F*, lower panel). Owing to the TMT multiplexing strategy, in which paired tumors and NATs were analyzed in the same batches, the *N*-glycans identified on the same proteins and the glycans detected at the same *N*-glycosylation sites were largely consistent between tumors and NATs. However, the abundance of many *N*-glycopeptides differed between tumors and NATs. This suggests that the observed differences reflect intrinsic tissue characteristics rather than experimental variability. All identified *N*-glycopeptides conformed to the canonical *N*-glycosylation sequon N-X-S/T/C, where X represents any amino acid except proline. Statistical analysis revealed that the N-X-T motif (53.1%) was more prevalent than N-X-S (41.8%), while N-X-C accounted for ∼5.1% of all sites (Fig. 1*G*). To investigate microheterogeneity of *N*-glycans between tumors and NATs, we categorized the glycan structures into five groups based on a literature-based classification (40): mannose, sialylated, fucosylated, complex/hybrid, and fucosylated-sialylated glycans. Mannose was the most predominant glycan type, followed by fucosylated glycans (Fig. 1*H*). High-mannose type glycans are widely distributed in the human body and are involved in essential biological functions, including cellular migration, intercellular communication, and immune recognition (46, 47, 48). In CCA, high-mannose glycans have been implicated in facilitating tumor spread and are being explored as therapeutic targets (49). We also analyzed the co-occurrence of the five glycan types on individual *N*-glycosylation sites. The majority of *N*-glycosylated sites contained a single glycan type, with mannose being the most predominant. Additionally, fucosylated and fucosylated-sialylated glycans were the most frequently co-occurring glycan types at the same *N*-glycosylation sites, suggesting potential functional relevance in eCCA (Fig. 1*I*). Our findings provide a comprehensive characterization of the eCCA glycoproteome, revealing the structural complexity of tumor-associated *N*-glycans.

### *N*-Glycoproteomic Differences Between eCCA and iCCA

**Fig. 2:**
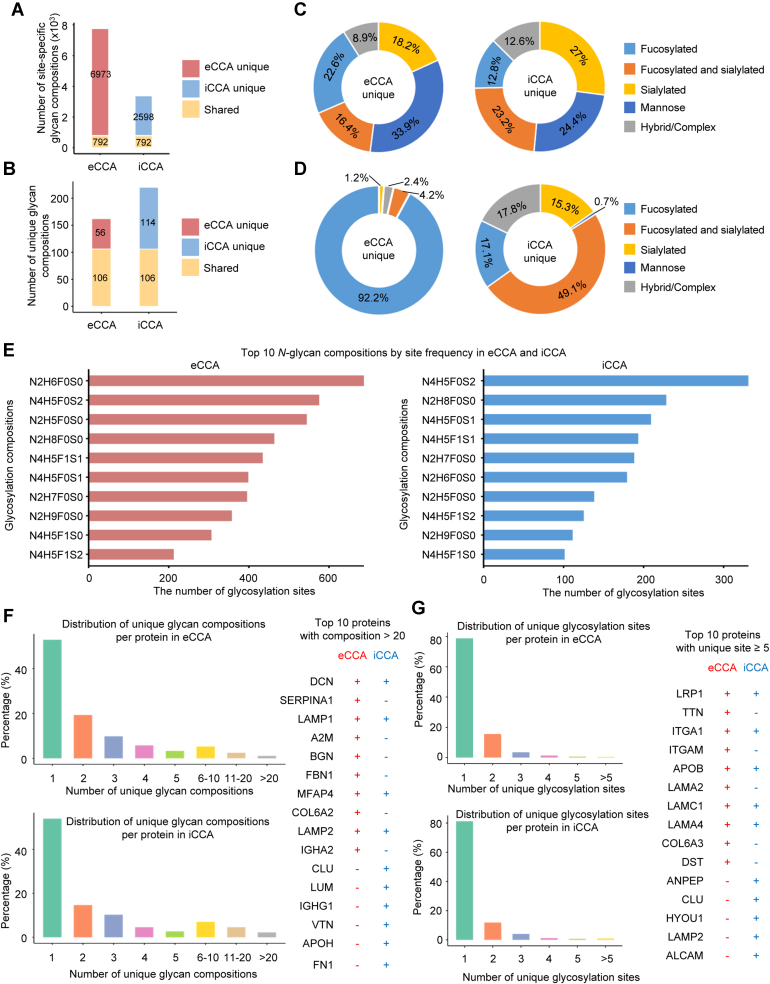
**Glycan comparison between eCCA and iCCA.***A-B*, bar plot showing the number of site-specific glycan compositions (*A*) and unique glycan compositions (B) specific to eCCA, specific to iCCA, or shared by both subtypes. *C*, distribution of site-specific glycan compositions in eCCA (*left*) and iCCA (*right*), showing the proportions of five glycan types. *D*, distribution of unique glycan compositions in eCCA (*left*) and iCCA (*right*), categorized by glycan structural features. Different colors represent distinct glycoform categories, as indicated in the legend. *E*, top 10 glycans with the highest number of *N*-glycosylation sites in eCCA (*left*) and iCCA (*right*). Bars represent *N*-glycosylation compositions ranked by site frequency. *F-G*, distribution of unique *N*-glycan compositions (*F*) and unique *N*-glycosylation sites (*G*) per protein in eCCA and iCCA. The top 10 *N*-glycoproteins enriched in unique *N*-glycan compositions and *N*-glycosylation sites are listed, with differences observed between tumor subtypes. eCCA, extrahepatic cholangiocarcinoma; iCCA, intrahepatic cholangiocarcinoma.

**Supplementary Fig. S1:**
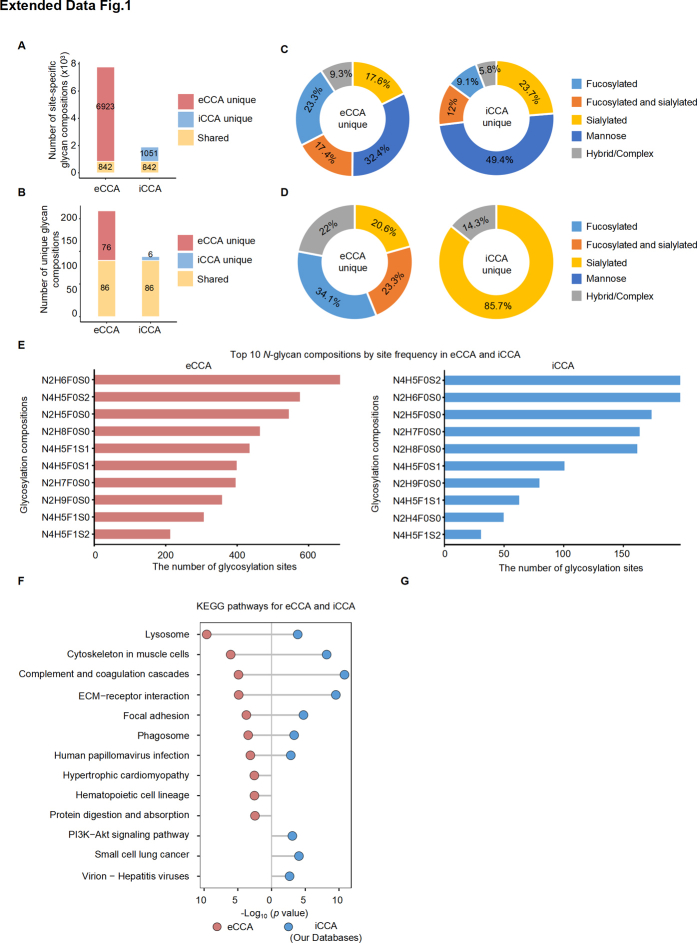
Comparative analysis of *N*-glycosylation profiles between eCCA and iCCA based on our in-house datasets. *A–B*, Bar plots showing the number of site-specific (*A*) and unique (*B*) glycan compositions that are specific to eCCA, specific to iCCA, or shared between the two subtypes. *C–D*. Proportional distribution of glycan types in eCCA and iCCA, based on site-specific compositions (*C*) and unique glycan compositions (*D*), categorized by structural features. *E*. Top 10 most frequent N-glycan compositions ranked by the number of glycosylation sites in eCCA (*left*) and iCCA (*right*). *F*. KEGG pathway enrichment of glycoproteins carrying subtype-specific glycopeptides, illustrating distinct functional associations between eCCA and iCCA.

To explore the *N*-glycosylation difference across CCA subtypes, we compared eCCA *N*-glycoproteomic dataset with a publicly available iCCA *N*-glycoproteomic dataset (19). To characterize *N*-glycosylation heterogeneity across subtypes, we analyzed site-specific glycan compositions, which represent defined glycan compositions at individual glycosylation sites, and unique glycan compositions, independent of their protein or site context. A total of 6973 site-specific glycan compositions were exclusively identified in eCCA, whereas 2598 were unique to iCCA, with 792 shared between the two subtypes (Fig. 2*A* and supplemental Table S4). Similarly, the analysis of unique glycan compositions revealed 56 unique glycan compositions in eCCA and 114 in iCCA, with 106 glycan compositions shared (Fig. 2*B*). Analysis of site-specific glycan compositions showed that eCCA exhibited a significantly higher proportion of fucosylated glycans (22.6%) and mannose glycans (33.9%), whereas iCCA was predominantly enriched in sialylated glycans (27%) (Fig. 2*C*). This trend was supported by the unique glycan composition analysis, in which 92.2% of the unique glycans in eCCA were fucosylated, while iCCA displayed a greater proportion of sialylated (15.3%) and fucosylated-sialylated (49.1%) glycans (Fig. 2*D*). These findings indicate that iCCA is associated with a higher proportion of sialylated glycans, whereas eCCA exhibits a predominance of fucosylated glycans. The distinct *N*-glycosylation signatures between eCCA and iCCA reflect subtype-specific molecular mechanisms and yield insights into potential glycosylation-associated biomarkers and therapeutic targets for each subtype. We further identified the top 10 most abundant glycan compositions in eCCA and iCCA (Fig. 2*E*). The overall glycosylation profiles were similar between the two subtypes, differing primarily in the rank order of glycans. Notably, the glycan composition N4H5F0S2, indicative of a complex glycan with four HexNAc, five Hexose, no Fucose, two Sialic acids, was highly prevalent in iCCA, whereas N2H6F0S0, a high-mannose glycan, was more enriched in eCCA. We then evaluated the distribution of *N*-glycosylation modifications across glycoproteins in each subtype. The proportions of proteins with varying numbers of unique glycans were similar between eCCA and iCCA: in both, over half of glycoproteins carried only one unique glycan, and fewer than 5% had more than 10 unique glycans (Fig. 2*F*). However, the specific glycoproteins exhibiting high glycan heterogeneity differed by subtype. In eCCA, proteins such as SERPINA1, A2M, BGN, FBN1, COL6A2, and IGHA2 had especially high glycoform diversity, whereas in iCCA, CLU, LUM, IGHG1, VTN, APOH, and FN1 showed greater diversity. Some proteins (e.g., DCN, LAMP1, MFAP4, LAMP2) were among the most highly glycosylated in both subtypes, indicating certain *N*-glycosylation complexities are shared. We also compared the number of *N*-glycosylation sites per protein between subtypes (Fig. 2*G*). Among the most extensively glycosylated proteins, LRP1, ITGA1, APOB, LAMC1, and LAMA4 had a high number of *N*-glycosylation sites in both subtypes. In contrast, certain glycoproteins exhibited marked differences in the number of detected glycosylation sites between eCCA and iCCA. Specifically, ANPEP, CLU, and HYOU1 harbored relatively more glycosylation sites in iCCA, whereas TTN, ITGAM, and LAMA2 showed greater glycosylation in eCCA. These observations suggest that while many *N*-glycoproteins undergo extensive *N*-glycosylation in both subtypes, some exhibit distinct site-specific *N*-glycosylation patterns, potentially reflecting different roles or regulatory mechanisms between eCCA and iCCA. In addition, to further validate the glycan composition trends between iCCA and eCCA, we performed *N*-glycoproteomic profiling on five additional paired iCCA samples using the same enrichment strategy and database search parameters. A total of 1968 glycopeptides, 1134 glycosylation sites, and 937 glycoproteins were identified (Supplemental Table S4). The results similarly demonstrate that eCCA exhibits a higher proportion of fucosylated glycans, whereas iCCA shows increased levels of sialylated glycans (Supplemental Fig. S1, *A–D*), consistent with our previous findings based on public dataset comparisons. Moreover, both subtypes share major glycan compositions that align with earlier comparative analyses (Supplemental Fig. S1*E*). Taken together, our results reveal distinct *N*-glycosylation landscapes between eCCA and iCCA. These differences may contribute to their unique roles in tumor progression, microenvironment interactions, and therapeutic responses.

**Fig. 3:**
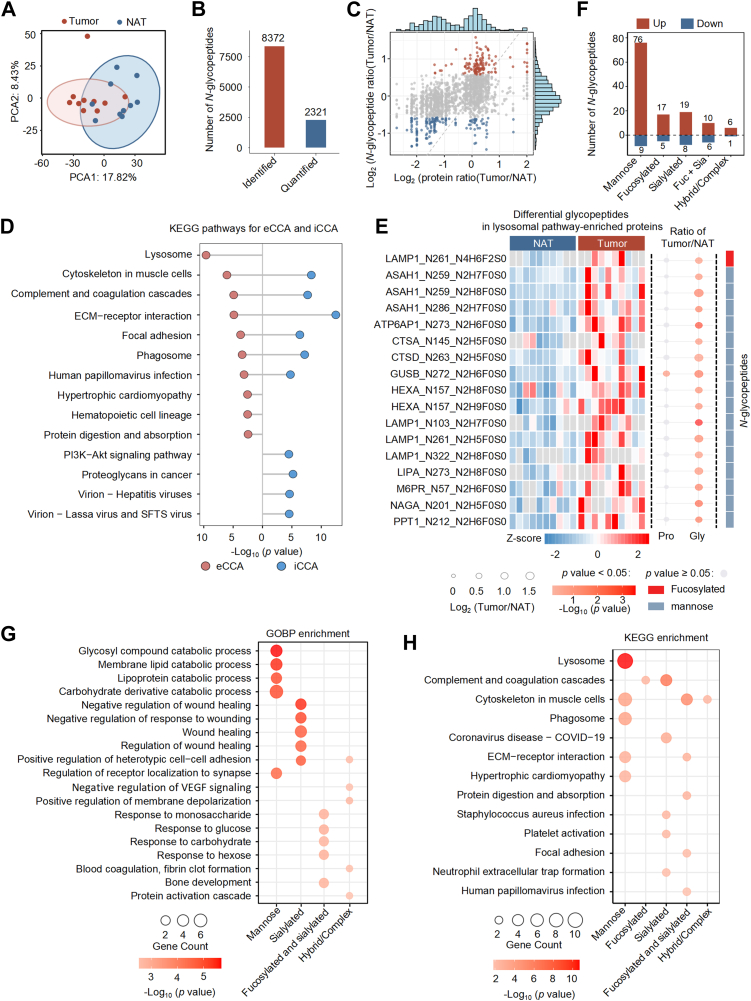
**Comparison of differential *N*-glycoproteins and associated pathways between eCCA and iCCA.***A*, PCA plot of *N*-glycopeptides in tumors and NATs. *B*, the number of *N*-glycopeptides identified; and the number reliably quantified (present in at least 50% of samples). *C*, histogram and scatter plot of log_2_*N*-glycopeptide ratios (Tumor/NAT) compared to log_2_ protein ratios. *D*, top 10 enriched KEGG pathways in eCCA and iCCA. The dot plot highlights the most significantly enriched pathways for eCCA (*red*) and iCCA (*blue*). *E*, differential glycopeptides in lysosomal pathway-enriched proteins. The heatmap shows *N*-glycopeptide expression in NAT and Tumor samples. The *right panel* indicates the Tumor/NAT ratio, with dot sizes representing log2 fold change (Log2FC). Colors denote significant (*p* < 0.05) and non-significant (*p* ≥ 0.05, *gray*) glycopeptides. *F*, classification of upregulated and downregulated *N*-glycopeptides based on glycan type. *G-H*, enrichment analysis of differentially glycosylated proteins in eCCA, including GOBP (*G*) and KEGG pathways (*H*). eCCA, extrahepatic cholangiocarcinoma; iCCA, intrahepatic cholangiocarcinoma.

Next, we investigated the *N*-glycosylation alterations within eCCA. Principal component analysis (PCA) revealed a partial separation between eCCA tumors and NATs based on *N*-glycosylation profiles, with PCA1 and PCA2 explaining 26.3% of the variance (Fig. 3*A*). A total of 2321 glycopeptides were quantified in more than 50% of the samples and used for subsequent differential analysis (Fig. 3*B* and supplemental Table S5). We then examined differentially expressed *N*-glycopeptides. Notably, the majority of *N*-glycopeptide changes occurred independently of their corresponding protein expression (Fig. 3*C*). To elucidate the functional implications of altered *N*-glycosylation and to compare the differences between iCCA and eCCA, we performed KEGG enrichment analysis (Fig. 3*D* and supplemental Table S6). *N*-Glycopeptides upregulated in tumors in each subtype were significantly enriched in pathways related to cytoskeletal organization, complement and coagulation cascades, ECM–receptor interaction, focal adhesion, and phagosome formation (Fig. 3*D*). This pattern was similarly observed in our own iCCA dataset, with most enriched pathways showing strong concordance with those identified using public datasets (Supplemental Fig. S1*F*). A comparable degree of consistency was also observed in comparisons with eCCA, providing robust cross-validation of our findings. These commonly enriched pathways highlight shared glycosylation-associated mechanisms underlying tumor progression in both subtypes. Notably, eCCA exhibited stronger enrichment in lysosome-related pathways, suggesting potential subtype-specific differences in glycoprotein trafficking and degradation. Several glycoproteins associated with the lysosomal pathway, including LAMP1, ASAH1, ATP6AP1, and HEXA, exhibited significantly increased levels of specific glycoforms bearing high-mannose glycans in tumor tissue (Fig. 3*E*). Importantly, these *N*-glycosylation changes were independent of protein expression, and most of the attached glycans were of the high-mannose type. These findings suggest that mannose-enriched glycoforms may be associated with lysosome-related glycoproteins in eCCA, potentially reflecting alterations in lysosomal pathways. To further refine the role of different glycan types in eCCA, we classified the differentially expressed *N*-glycopeptides into five categories. The results revealed that mannose-type glycans were the most predominant, with 76 upregulated and nine downregulated *N*-glycopeptides (Fig. 3*F*). We then performed Gene Ontology Biological Process (GOBP) and KEGG pathway enrichment analyses for differentially *N*-glycosylated proteins in eCCA, grouped by glycan types (Fig. 3, *G* and *H* and supplemental Table S6). *N*-Glycoproteins carrying high-mannose glycan alterations were significantly enriched in pathways related to glycan metabolism, receptor signaling, lysosomal function, and ECM remodeling. In contrast, proteins with increased sialylated peptides were associated with pathways in wound healing, complement and coagulation cascades, and platelet activation, implicating these modifications in modulating the tumor microenvironment and immune responses. These findings highlight that aberrant *N*-glycosylation in eCCA may affect key oncogenic pathways and suggest that specific glycan modifications play distinct roles in eCCA pathogenesis.

### Aberrant *N*-Glycosylation is Associated With Immunosuppression in eCCA

**Fig. 4:**
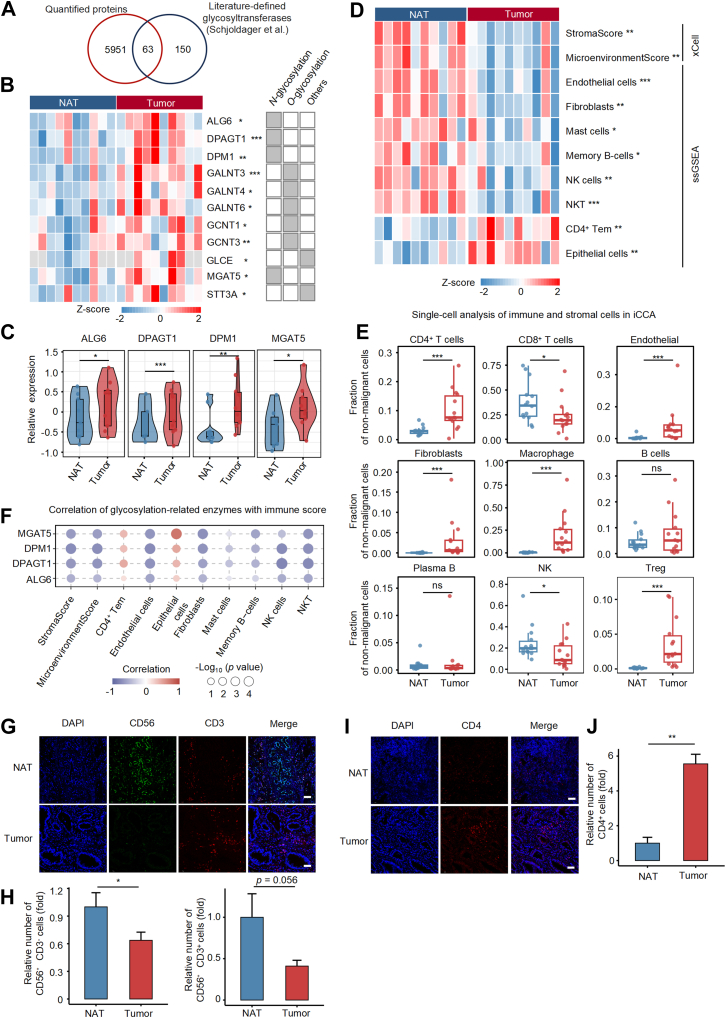
**Immune signatures in iCCA and eCCA and associated glycosyltransferases.***A*, Venn diagram showing the overlap between quantified proteins and glycosyltransferases reported by Schjoldager *et al*., identifying 63 glycosyltransferases in eCCA samples. *B*, heatmap of differentially expressed glycosyltransferases in eCCA tumors versus NATs. *C*, Violin plots comparing the expression of selected glycosyltransferases (ALG6, DPAGT1, DPM1, MGAT5) between tumors and NATs. *D*, heatmap of immune cell infiltration in eCCA tumors *versus* NATs based on xCell and ssGSEA scoring. *E*. Single-cell RNA-seq analysis of immune and stromal cell populations in iCCA (data from Song *et al*., 2022), comparing cell fraction changes between iCCA tumors and NATs. *F*, correlation analysis between the expression of glycosylation-related enzymes and immune cell infiltration. *G*, representative immunofluorescence staining of NAT and tumor tissues from five paired eCCA samples. Dual immunofluorescence staining was performed using DAPI (*blue*), CD56 (*green*), and CD3 (*red*); individual channels and merged images are shown. *H*, quantification of CD56^+^CD3^+^ NKT cells and CD56^+^CD3^-^ NK cells, expressed as relative abundance (fold change normalized to NAT). *I*, representative staining and quantification of CD4^+^ T cells (*red*) by single-marker immunofluorescence. *J*. Quantification of CD4^+^ T cells show a significant increase in tumor tissues compared to NAT. Scale bars: 50 μm. Statistical significance was assessed using the Mann–Whitney *U* test, ∗*p* < 0.05; ∗∗*p* < 0.01; ∗∗∗*p* < 0.001. eCCA, extrahepatic cholangiocarcinoma; iCCA, intrahepatic cholangiocarcinoma; ssGSEA, single sample gene set enrichment analysis.

*N*-glycosylation, in which oligosaccharides are attached to Asn in N-X-S/T/C motifs (X ≠ Pro), involves a series of enzymes responsible for glycan initiation, elongation, and branching (40, 50, 51). To further explore *N*-glycosylation alterations in eCCA, we analyzed glycosyltransferases curated by Schjoldager *et al* (52). Among 6014 quantified proteins, 63 were glycosyltransferases, with 11 significantly changed in tumor tissues compared to NATs (Fig. 4, *A* and *B*). Notably, four key *N*-glycosylation enzymes, ALG6, DPAGT1, DPM1, and MGAT5, were significantly upregulated in eCCA tumors (Fig. 4*C*), suggesting their involvement in *N*-glycan remodeling in this subtype. It is worth noting that DPM1 is known to promote tumor progression and may serve as a prognostic biomarker in hepatocellular carcinoma (53, 54). MGAT5, responsible for adding β1,6-GlcNAc branches to *N*-glycans, can promote immune evasion and oncogenic signaling in multiple solid tumors, including pancreatic, ovarian, and breast cancers (55, 56, 57, 58). Given the well-established role of glycosylation in modulating T cell activation and immune tolerance (14, 59, 60), we next characterized the TME in eCCA using ssGSEA and xCell (42). Compared to NATs, eCCA tumors showed significantly reduced stromal and immune scores, indicating a less infiltrated, more immunosuppressive TME (Fig. 4*D*). Immune profiling revealed decreased infiltration of NK cells, NKT cells, endothelial cells, fibroblasts, mast cells, and memory B cells, while epithelial cells and CD4^+^ Tem were increased. These observations were further validated by a single-cell transcriptomic results that identified 11 major cell subtypes and confirmed reduced endothelial cells and fibroblasts, and increased epithelial cells in eCCA (61). To assess differences between eCCA and iCCA, we analyzed publicly available scRNA-seq data (62). Both subtypes exhibited reduced NK cells in tumors, but iCCA tumors uniquely showed higher fibroblast and endothelial cell content than eCCA, suggesting distinct TME remodeling mechanisms (Fig. 4*E*). Besides, we performed correlation analyses between glycosyltransferase expression and immune cell scores in eCCA. ALG6, DPAGT1, DPM1, and MGAT5 were significantly negatively correlated with stromal and immune scores, and with multiple immune and stromal cell populations, including NK cells, NKT cells, endothelial cells, fibroblasts, and memory B cells (Fig. 4*F* and supplemental Table S7). To further validate our immune cell estimation based on proteomics and immune gene signatures, we examined the infiltration of CD56^+^CD3^-^ NK cells, CD56^+^CD3^+^ NKT cells, and CD4^+^ T cells in five paired eCCA tumor and adjacent normal tissues by immunofluorescence staining. CD56^+^CD3^-^ NK cells and CD56^+^CD3^+^ NKT cells were markedly reduced in tumor tissues compared to NAT, with the decrease in NK cells reaching statistical significance (*p* < 0.05), while NKT cells showed a similar decreasing trend (*p* = 0.056) (Fig. 4, *G* and *H*). In contrast, the proportion of CD4^+^ T cells was significantly elevated in tumor tissues (Fig. 4, *I* and *J*). These spatial findings are consistent with the proteomic and computational immune estimates, further supporting the reliability of our immune profiling approach. These data support a role for aberrant glycosylation in shaping an immunosuppressive, stroma-depleted TME in eCCA, highlighting glycosylation pathways as potential targets to enhance anti-tumor immunity.

### DPM1-Associated *N*-Glycosylation Reflects Immune Microenvironment Alterations in eCCA

**Fig. 5:**
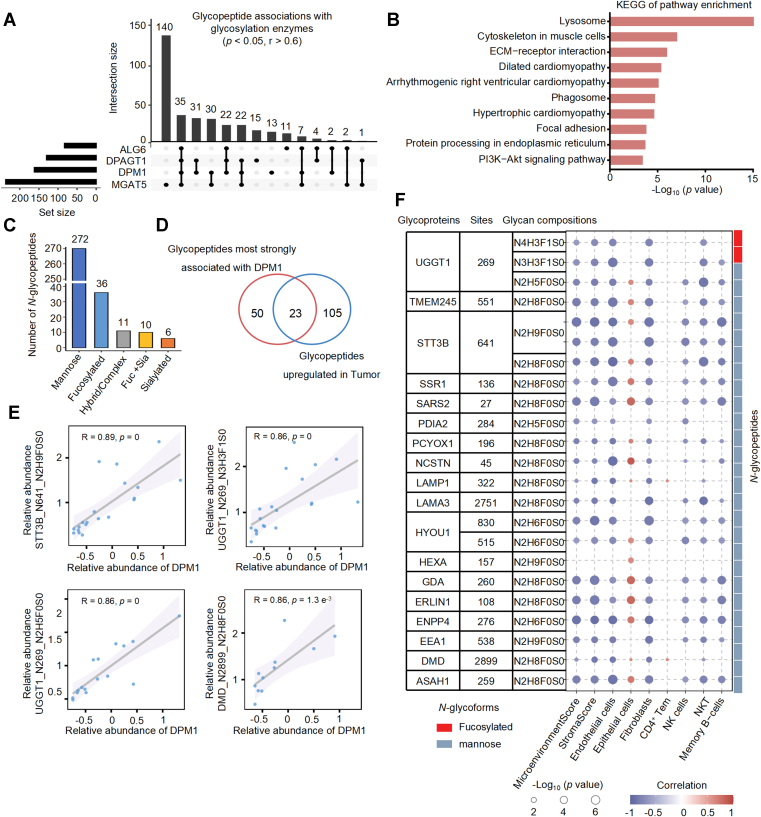
**DPM1 potentially links *N*-glycosylation to immune regulation.***A*, UpSet plot depicting *N*-glycopeptides significantly correlated with four key glycosylation enzymes (ALG6, DPAGT1, DPM1, and MGAT5) based on Spearman correlation analysis (*p* < 0.05, correlation >0.6). *B*. Top 10 enriched KEGG pathways for glycopeptides significantly correlated with glycosylation enzymes. *C*, classification of glycopeptides significantly associated with the four glycosylation enzymes, with mannose-type glycans being the most enriched category. *D*, Venn diagram showing overlap between glycopeptides most strongly correlated with DPM1 and those upregulated in tumor tissues. *E*, representative scatter plots illustrating strong positive correlations between DPM1 expression and *N*-glycopeptide abundance. *F*, heatmap showing the correlation between 23 DPM1-associated *N*-glycopeptides and immune cell infiltration. Circle size represents the -log10(*p*-value), while color indicates the correlation coefficient with red for positive and *blue* for negative correlations. The *red* box highlights fucosylated glycoforms, and the *blue* box indicates mannose-containing glycoforms.

To identify key regulators of the altered N-glycoproteome in eCCA, we examined correlations between glycopeptide abundance and glycosylation enzyme expression levels. DPM1, MGAT5, DPAGT1, and ALG6 showed significant associations (correlation coefficient >0.6, *p* < 0.05) with distinct glycopeptide subsets (Fig. 5*A*). KEGG pathway enrichment of glycoproteins corresponding to these glycopeptides revealed significant enrichment in lysosome-related and cell adhesion-related signaling pathways (Fig. 5*B*). Glycan classification of all glycopeptides significantly associated with these enzymes indicated a predominance of mannose-type structures (Fig. 5*C*). As is well established, DPM1 is a key enzyme responsible for the synthesis of dolichol-phosphate-mannose, an essential mannose donor in the *N*-glycosylation pathway, and thus plays a central role in the generation of mannose-rich glycans (44). Given this functional role, we focused on DPM1 for downstream analyses. Integration of glycopeptide–DPM1 correlation data with tumor-versus-adjacent tissue differential expression identified 23 glycopeptides that were both strongly associated with DPM1 expression and significantly upregulated in tumor tissues (Fig. 5*D*). Representative glycopeptides from STT3B, UGGT1, and DMD exhibited strong positive correlations with DPM1 abundance (Fig. 5*E* and supplemental Table S8).

To assess potential immunological implications, we correlated the tumor-upregulated, DPM1-associated glycopeptides with tumor-infiltrating immune cell populations. Several high-mannose-type N-glycopeptides, such as UGGT1_N269_N2H5F0S0, STT3B_N641_N2H9F0S0, STT3B_N641_N2H8F0S0, HYOU1_N830_N2H6F0S0 and HYOU1_N515_N2H6F0S0, displayed significant associations with natural killer (NK) cells, natural killer T (NKT) cells, and memory B cells (Fig. 5*F*), suggesting that DPM1 may modulate mannose-rich glycosylation, thereby indirectly influencing immune cell dynamics within the eCCA microenvironment.

### DPM1 is Upregulated in eCCA and Linked to Mannose-type *N*-Glycosylation Remodeling and Cell Migration

**Fig. 6:**
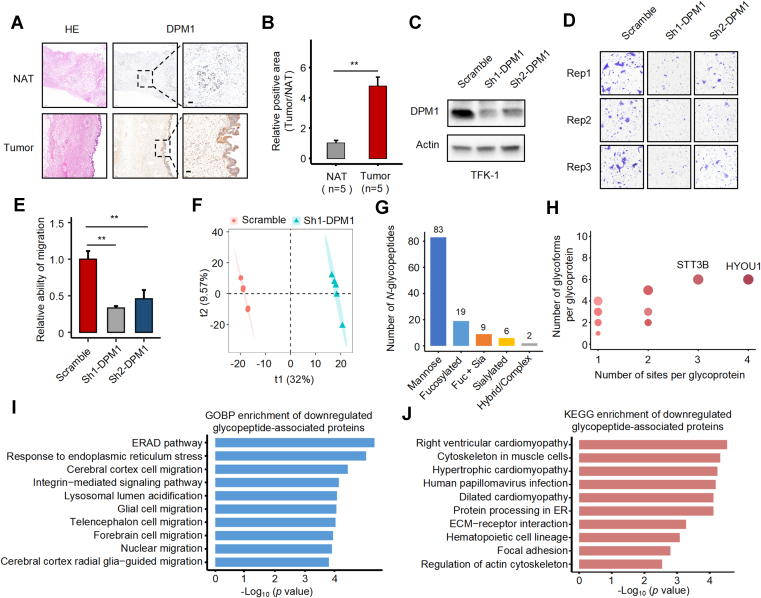
**DPM1 is upregulated in eCCA and is functionally associated with mannose-rich *N*-glycosylation changes and increased cell migration.***A*, representative hematoxylin and eosin (HE) staining and DPM1 immunohistochemistry (IHC) in paired tumor and NAT tissues from eCCA patients. *B*, quantification of DPM1 IHC signals as relative positive area (Tumor/NAT) in n = 5 paired samples (*p* < 0.01 by Mann-Whitney U test). *C*. Western blot validation of DPM1 knockdown efficiency in TFK-1 cells using two independent shRNAs. Actin served as a loading control. *D*, transwell migration assays performed in scramble and DPM1-depleted TFK-1 cells. *E*, relative migration ability was quantified and normalized to the scramble control. Each assay was performed in triplicate. Statistical significance between groups was assessed using a paired *t* test. ∗*p* < 0.05; ∗∗*p* < 0.01. *F*, partial least squares discriminant analysis (PLS-DA) showing distinct separation of *N*-glycopeptide profiles between scramble and shDPM1-treated cells. *G*, classification of significantly downregulated *N*-glycopeptides (n = 119; *p* < 0.05, FC < 0.8) upon DPM1 knockdown, highlighting the predominance of mannose-rich glycans. *H*, distribution of glycosylation density in downregulated glycoproteins, based on number of *N*-glycosites and glycoforms per protein. Bubble size corresponds to number of glycoforms. *I*, GO biological process (GOBP) enrichment analysis of glycoproteins corresponding to downregulated glycopeptides. *J*. KEGG pathway enrichment analysis of the same glycoprotein set, including several immune-related pathways such as hematopoietic cell lineage.

To investigate the functional role of DPM1 in eCCA, we performed immunohistochemical staining on paired tumor and adjacent normal tissues. DPM1 expression was significantly elevated in tumor regions, as confirmed by increased positive staining area (Fig. 6, *A* and *B*). Western blot analysis confirmed efficient knockdown of DPM1 using two independent shRNAs in TFK-1 cells (Fig. 6*C*). Functionally, DPM1 depletion led to a marked reduction in cell migratory capacity in transwell assays (Fig. 6, *D* and *E*), supporting a promigratory role of DPM1 in eCCA progression. To investigate glycoproteomic alterations associated with DPM1, we conducted tandem mass tag (TMT)-based quantitative *N*-glycoproteomic profiling in TFK-1 cells, identifying 1172 *N*-glycopeptides, 565 *N*-glycosites, and 412 *N*-glycoproteins (Supplemental Table S9). Partial least squares discriminant analysis (PLS-DA) revealed a clear separation in glycopeptide profiles between DPM1-depleted and control cells (Fig. 6*F*). A total of 119 glycopeptides were significantly downregulated following DPM1 knockdown, with the majority (69.7%) carrying mannose-rich structures (Fig. 6*G*). Downregulated glycopeptides were mapped to glycoproteins with high site density and glycoform diversity, including HYOU1 and STT3B (Fig. 6*H*). Gene Ontology analysis of these glycoproteins revealed enrichment in ER stress, lysosomal acidification, and cell–matrix adhesion pathways (Fig. 6*I*). KEGG pathway enrichment revealed multiple affected signaling pathways, including protein processing in the ER, ECM–receptor interaction, and hematopoietic cell lineage. These pathways collectively suggest that DPM1 may influence glycosylation indirectly by modulating the availability of mannose donors, thereby impacting cellular adhesion, protein homeostasis, and immune cell function (Fig. 6*J*).

## Discussion

In summary, we present the *N*-glycoproteomic landscape of eCCA and perform an in-depth comparison of *N*-glycoproteins, *N*-glycosites, glycan compositions, and enriched pathways between eCCA and iCCA. Our findings revealed that eCCA is characterized by an enrichment of fucosylated glycans, while iCCA exhibits higher levels of sialylation. Further association analysis revealed the connection between *N*-glycosylation and immune regulation. Notably, aberrant *N*-glycosylation in eCCA was associated with the upregulation of certain glycosylation enzymes such as ALG6, DPAGT1, DPM1, and MGAT5. Among them, DPM1, through its role in mannose donor biosynthesis, may indirectly modulate *N*-glycosylation patterns linked to an immunosuppressive tumor microenvironment, underscoring its involvement in eCCA pathogenesis and potential as a candidate for targeted therapy.

Effective glycopeptide enrichment is critical for MS-based glycoproteomics, as glycopeptides are typically scarce and highly heterogeneous in biological samples. Various enrichment strategies have been developed to address this challenge, such as lectin affinity, hydrazide chemistry, boric acid-based methods, mixed anion-exchange (MAX), and HILIC (63). In this study, ZIC-HILIC was used for glycopeptide enrichment in the eCCA glycoproteomic dataset, whereas the iCCA data from Li *et al*. employed the MAX enrichment approach. ZIC-HILIC and MAX exhibit differences to some extent in glycopeptide enrichment performance. Recent studies have shown that ZIC-HILIC outperforms MAX, achieving a 26% increase in identified glycopeptides, along with broader glycan coverage and higher enrichment efficiency. Specifically, ZIC-HILIC preferentially enriches high-mannose and sialylated glycans, particularly those with higher molecular weights, making it a more effective and less biased approach for comprehensive glycoproteomic profiling (64). Differences in enrichment strategies and data processing may partly explain the discrepancies between datasets, so glycan-type comparisons between iCCA and eCCA should be interpreted with caution. To reduce platform-related bias, we avoided relying on absolute abundance or direct peptide-level overlap, and instead focused on glycan-type trends and tumor–normal differences. We also standardized downstream analysis, including glycan classification and enrichment, to ensure consistency. Despite potential technical influences, our findings provide valuable insights into subtype-specific glycosylation patterns in cholangiocarcinoma.

The relatively higher levels of fucosylation observed in eCCA compared to iCCA may result from a shift in terminal glycan modifications rather than changes in the dominant glycoform type. This increase in fucosylation likely reflects subtype-specific differences in glycosyltransferase expression, Golgi function, or microenvironmental cues, similar to the higher sialylation observed in iCCA. As terminal glycan modifications, both sialylation and fucosylation are known to regulate cell–cell interactions, tumor invasion, metastasis, and immune responses (15, 65, 66). Notably, high-mannose glycans represent the predominant glycoform in eCCA, both globally and in tumor-versus-NAT comparisons. Their enrichment in eCCA may indicate impaired glycan maturation or altered Golgi processing (67). Recent studies have showed that metastasis of CCA is driven by the accumulation of extended high-mannose glycans, particularly α-1,2-mannosylated *N*-glycans, which promote cell migration and may serve as potential therapeutic targets (49). This distinction reconciles the elevated fucosylation in eCCA relative to iCCA with the predominance of high-mannose glycans in tumors, highlighting the complexity of subtype-specific glycoproteomic reprogramming.

Functional annotation of tumor-enriched *N*-glycosylated proteins in eCCA revealed that lysosome-related pathways were the most significantly affected by *N*-glycosylation. As a central organelle for cellular degradation and proteostasis, the lysosome has emerged as a critical regulator in cancer biology. In CCA, impaired lysosome biogenesis is associated with increased exosome secretion and enhanced metastatic potential (68). The consistent enrichment of lysosome-associated pathways supports the involvement of lysosomes in CCA tumorigenesis through modulation of intracellular degradation and metabolic adaptation (69). These findings underscore the critical role of lysosomes in CCA progression; however, the function of lysosomal glycosylation in eCCA remains largely unexplored. Previous studies have demonstrated that lysosomal membrane protein LAMP2, glycosylated by MGAT5, promotes choriocarcinoma progression by enhancing *N*-glycan–mediated cell adhesion through galectin interactions (70). In addition, LAMP1 glycosylation exhibits tumor-specific alterations in pancreatic (71) and breast cancers (72, 73), influencing extracellular matrix interactions, drug response, and tumor heterogeneity, suggesting its potential as a biomarker and functional modulator in cancer. Therefore, the glycosylation of these lysosomal proteins, particularly on proteins such as LAMP1 and ASAH1, may play a critical role in the pathogenesis of eCCA. Our findings highlight lysosomal glycoproteins as promising candidates for further investigation in eCCA, with their glycosylation patterns potentially offering novel insights into tumor progression mechanisms and therapeutic targeting.

One of the key findings of this study is the identification of DPM1 as a central glycosylation enzyme associated with tumor-specific glycopeptides and reduced immune cell infiltration in extrahepatic cholangiocarcinoma (eCCA). DPM1 functions as the catalytic subunit of the dolichol-phosphate-mannosyltransferase (DPMS) complex, which is essential for the biosynthesis of mannose donors required for multiple glycosylation pathways, including *N*-glycosylation, O- and C-mannosylation, and glycosylphosphatidylinositol (GPI) anchor formation (44, 74, 75). In eukaryotes, Dol-P-Man is synthesized on the cytosolic side of the ER and flipped into the lumen to fuel glycosylation, linking sugar metabolism to protein folding and ER homeostasis (76). Although DPM1 does not directly catalyze glycan transfer, it plays a fundamental role in regulating protein glycosylation through the modulation of donor substrate availability. Previous studies have shown that DPM1 can influence biological processes such as desmosomal adhesion and epidermal differentiation via SERPINB5 (77). Moreover, DPM1 is upregulated in several cancers, including hepatocellular carcinoma and breast cancer, where it correlates with tumor progression, poor prognosis, and subtype-specific glycosylation features, particularly in luminal breast cancer (54, 78). In infectious diseases, DPM1-mediated GPI biosynthesis contributes to immune evasion by supporting the synthesis of immunomodulatory glycolipids (74). In the present study, we demonstrate that DPM1 is upregulated in eCCA and associated with altered glycosylation profiles that may contribute to an immunosuppressive tumor microenvironment. Collectively, these findings highlight DPM1 as a clinically relevant regulator of tumor immune escape, with potential as both a predictive biomarker and therapeutic target to improve immunotherapy outcomes in cholangiocarcinoma.

LacdiNAc (GalNAcβ1-4GlcNAc) is a disaccharide motif found on N- and O-glycans, formed by the addition of N-acetylgalactosamine (GalNAc) to N-acetylglucosamine (GlcNAc) via a β1-4 linkage. Although LacdiNAc modifications are relatively rare, they have been implicated in diverse biological processes, including cancer, aging, and immune regulation (79, 80, 81). A recent glycoproteomic study identified 113 LacdiNAc-modified glycopeptides from 33 glycoproteins, revealing that LacdiNAc-containing N-glycans are specifically upregulated in iCCA but not in hepatocellular carcinoma (HCC), underscoring their potential as iCCA-specific biomarkers and therapeutic targets (19). However, the role of LacdiNAc in eCCA remains unknown. In our dataset, among the 8372 glycopeptides identified in eCCA, only 15 carried LacdiNAc structures. After applying a 50% missing value threshold, only one LacdiNAc-bearing glycopeptide remained for differential analysis, and it did not reach statistical significance (Supplemental Table S2; glycan linkage annotation includes “41V”). Whether this striking difference reflects a true biological divergence between iCCA and eCCA or results from technical factors—such as differences in sample composition, detection sensitivity, or glycopeptide enrichment preferences—remains to be explored. Future studies focusing on LacdiNAc in cholangiocarcinoma would benefit from standardized analytical workflows to enable more accurate cross-subtype comparisons.

Despite these advances, our study has several limitations. First, the relatively small sample size may limit statistical power and the generalizability of the findings. Validation in larger, independent cohorts, along with functional studies, will be necessary to confirm the roles of specific *N*-glycosylation and glycosyltransferases. Second, although *in vitro* DPM1 knockdown revealed phenotypic changes, the underlying mechanisms remain unclear, and validation *in vivo* and across multiple dimensions is still lacking. Additionally, differences in analytical platforms and search algorithms between datasets (e.g., eCCA *versus* previously published iCCA) may introduce bias, although we mitigated this by standardizing glycan classification criteria.

In conclusion, our study provides a comparative analysis of *N*-glycosylation signatures between eCCA and iCCA and identifies DPM1 as a key regulator of tumor-associated glycopeptides and immune suppression in eCCA, offering new insights into its pathogenesis. Future studies should aim to validate these findings and explore glycosylation-targeted strategies to enhance anti-tumor immunity in CCA.

## Data Availability

The mass spectrometry proteomics and *N-gly*coproteomics datasets have been submitted to the ProteomeXchange Consortium (http://proteomecentral.proteomexchange.org) through the iProX partner repository (82) under the dataset identifier PXD062333.

## Supplemental Data

This article contains supplemental data (19, 41, 52).

## Conflict of Interest

The authors declare that they do not have any conflicts of interest with the content of this article.
