# Supplementary material for: Comparative N-Glycoproteomics Reveals Subtype-Specific N-Glycosylation Signatures and Immune Associations in Cholangiocarcinoma
Source: Mol Cell Proteomics. 2025 Oct 7;24(11):101084. doi: 10.1016/j.mcpro.2025.101084 (PMC12615305; doi:10.1016/j.mcpro.2025.101084)
Supplement: Supplemental Information [file mmc10.docx]

*Supplemental information*

**Comparative *N*-Glycoproteomics Reveals Subtype-Specific *N*-Glycosylation Signatures and Immune Associations in Cholangiocarcinoma**

Zhili Xia^1,†^, Li Gao^2,†^, Meng Hu^2,†^, Yingjie Li^2,†^, Kexin Yu^2,†^, Ningzu Jiang^1^, Long Gao^1^, Yu Liu^2^, Ying Lu^2^, Yanxian Ren^1 3^, Chenjun Tian^1^, Yawen Lu^1^, Jindu Zhang^1 3^, Haiying Yu ^4^, Ping Yue^1 3 4^, Yanyan Lin^1 3 4^, Rou Zhang^2^, Yanqiu Gong^2,^* , Wenbo Meng^1 3 4,^*

^1^The First School of Clinical Medicine, Lanzhou University, Lanzhou 730030, China.

^2^National Clinical Research Center for Geriatrics and Department of General Practice, State Key Laboratory of Biotherapy, West China Hospital, Sichuan University, Chengdu, 610041, China.

^3^Department of General Surgery, the First Hospital of Lanzhou University, Lanzhou 730030, China

^4^Gansu Province Key Laboratory of Biological Therapy and Regenerative Medicine Transformation, Lanzhou, China

^†^These authors contributed equally to this work.

*Correspondence: mengwb@lzu.edu.cn (MD., PhD. Wenbo Meng); niqiu.er@163.com (Dr. Yanqiu Gong)

**Supplemental table legends**

**supplemental Table S1**. The clinical information of patients with extrahepatic cholangiocarcinoma (eCCA) and intrahepatic cholangiocarcinoma (iCCA).

**supplemental Table S2**. Quantitative summary of the eCCA glycoproteome, including 8,372 *N*-glycopeptides, 3,467 *N*-glycosites, and 2,627 *N*-glycoproteins identified. The human N-glycome database used for identification comprised 6,709 entries generated via a retrosynthetic strategy.

**supplemental Table S3**. List of 6,014 proteins identified in eCCA samples based on proteomic analysis, including 1,132 overlapping N-glycoproteins.

**supplemental Table S4**. List of 4,371 N-glycopeptides identified in intrahepatic cholangiocarcinoma (iCCA) samples reported by Li et al. Additionally, based on comparison with this public dataset, 6,973 site-specific glycoform compositions were identified exclusively in eCCA, 2,598 compositions were unique to iCCA, and 792 compositions were shared between the two subtypes. A total of 1,968 N-glycopeptides were also identified in our own iCCA dataset.

**supplemental Table S5**. List of 2,321 *N*-glycopeptides quantified in eCCA samples (with ≤50% missing values across samples).

**supplemental Table S6**. Differentially expressed *N*-glycopeptides in eCCA and iCCA. This includes 128 upregulated and 29 downregulated *N*-glycopeptides in eCCA tumors compared to NATs, and 112 upregulated *N*-glycopeptides in iCCA compared to NATs (data from Li et al.) (1) . This table also contains the raw data corresponding to Figure 3D–H.

**supplemental Table S7**. List of 213 glycosyltransferases curated from Schjoldager et al(2), and tumor microenvironment (TME)-related genes from Lin et al(3). This table also includes the raw visualization data for Figure 4B–F.

**supplemental Table S8**. List of *N*-glycopeptides significantly correlated with four key glycosylation enzymes—ALG6, DPAGT1, DPM1, and MGAT5—based on Spearman correlation analysis (*p* < 0.05, R > 0.6). It also includes the correlation matrix between 23 DPM1-associated, tumor-enriched glycopeptides and immune cell infiltration scores, as well as the raw data for Figure 5.

**supplemental Table S9**. List of 1,172 N-glycopeptides identified in shDPM1 TFK-1 cells, including 119 significantly downregulated N-glycopeptides.

**Reference:**

1. Li J, Zhao T, Li J, Shen J, Jia L, Zhu B, et al. Precision N-glycoproteomics reveals elevated LacdiNAc as a novel signature of intrahepatic cholangiocarcinoma. Mol Oncol. 2022;16(11):2135-52.

2. Schjoldager KT, Narimatsu Y, Joshi HJ, Clausen H. Global view of human protein glycosylation pathways and functions. Nat Rev Mol Cell Biol. 2020;21(12):729-49.

3. Lin J, Dai Y, Sang C, Song G, Xiang B, Zhang M, et al. Multimodule characterization of immune subgroups in intrahepatic cholangiocarcinoma reveals distinct therapeutic vulnerabilities. J Immunother Cancer. 2022;10(7).
